# Supplementary material for: Chromosome biorientation produces hundreds of piconewtons at a metazoan kinetochore
Source: Nat Commun. 2016 Oct 20;7:13221. doi: 10.1038/ncomms13221 (PMC5080440; doi:10.1038/ncomms13221)
Supplement: Supplementary Information — Supplementary Figures 1 - 4 [file ncomms13221-s1.pdf]

Supplementary Figure and Figure Legends

Supplementary Figure 1

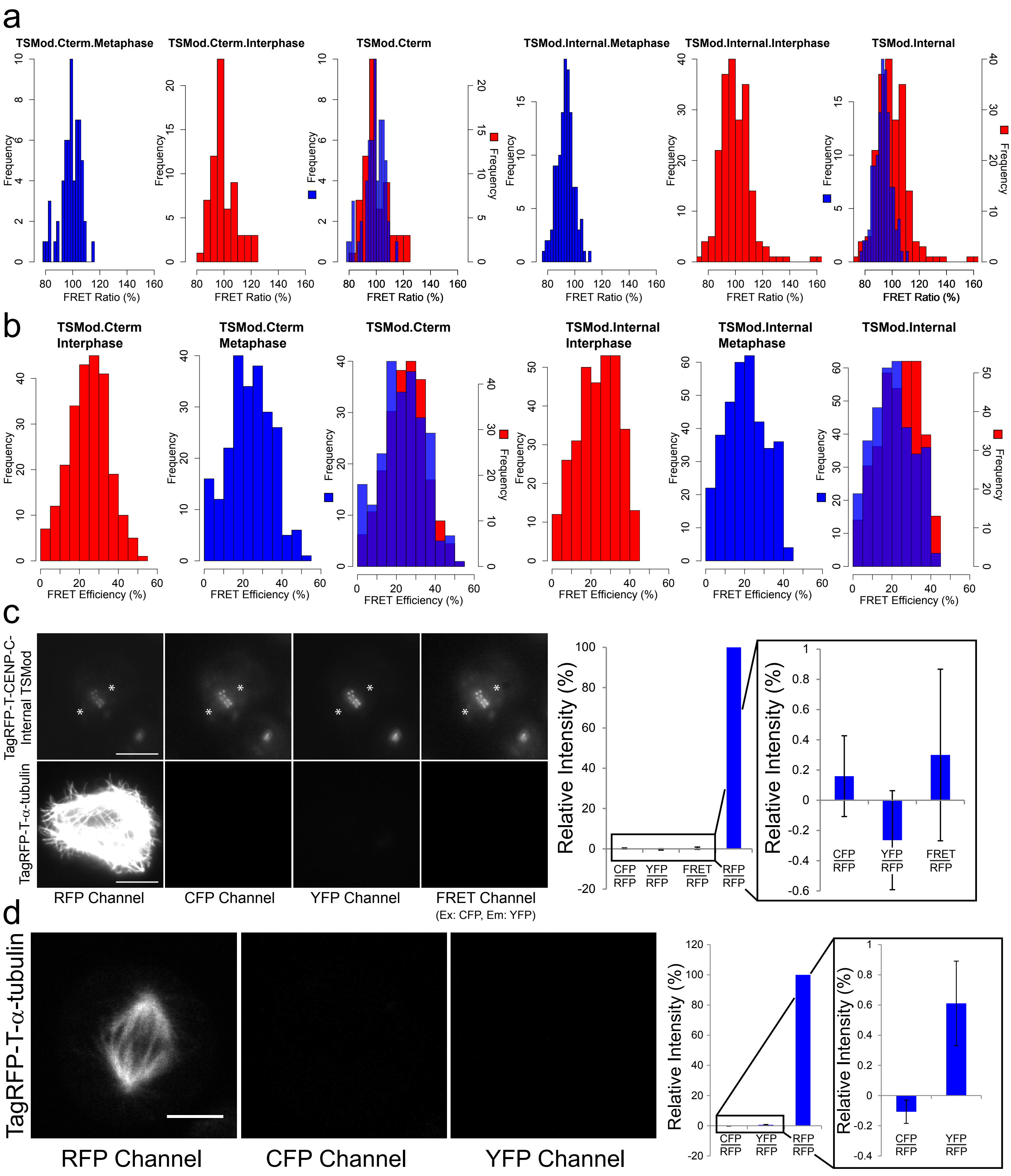

Supplementary Figure 1. (a) Histograms showing the distributions of normalized (to interphase condition for each reporter) FRET ratio measurements reported in Fig. 2d. (**b**) Histograms showing the distributions of the interphase and metaphase acceptor photobleaching data reported in Fig. 2f. All FRET data was analyzed by R. Shapiro-Wilk Normality test to determine if the data exhibited normal distributions. Other than the internal TSMod metaphase FRET ratio measurements and the C-TERM TSMod acceptor photobleaching data (interphase and metaphase), the data exhibited a non-normal distribution. Thus, pairwise p-values for the C-TERM acceptor photobleaching interphase and metaphase conditions were determined with the Student's t-test. All other p-values are from the Mann-Whitney-Wilcoxon test since these data exhibited non-normal distributions. (**c**) Measuring bleed-through from TagRFP-T-α-tubulin expressing cells on the microscope system used for the wide-field FRET imaging experiments reported in Fig. 2d. For comparison a TagRFP-T-CENP-C internal TSMod cell is shown that was imaged with the identical imaging parameters, and comparable channels are displayed with identical contrast and brightness scaling. Asterisks denote the approximate positon of the spindle poles. Bar graphs show quantifications of the spectral bleed-through from the TagRFP-T signal into the CFP, YFP, and FRET channels. The background corrected intensities of the CFP, YFP and FRET signals are reported as intensities relative to the background corrected RFP signal (set to 100). n = 34 TagRFP-T-labeled MT-containing regions from 7 cells. (**d**) Measuring bleed-through from TagRFP-T-α-tubulin expressing cells on the microscope system used for the acceptor photobleaching experiments reported in Figs. 2f, h. Representative TagRFP-T-α-tubulin expressing cell with the CFP and YFP channels imaged and scaled with identical parameters as the pre-bleached mTurquoise2 and mVenus channels shown in Fig. 2e. Bar graphs show quantifications of the spectral bleed-through from the TagRFP-T signal into the CFP, YFP channels. The background corrected intensities of the CFP, and YFP signals are reported as intensities relative to the background corrected RFP signal (set to 100). n = 11 cells.

The pre- and post-bleach CFP intensities are the only signals that are used to calculate the FRET efficiency and the YFP channel is imaged to confirm the efficiency of the acceptor photobleaching. Scale bars are 10 μm in (c) and 5 μm in (d). Error bars are SEM.

Supplementary Figure 2

Counting VH-EGFP per TagRFP-T-CENP-C-TR

1) Image TagRFP-T-CENP-C-EGFP: There is the same number of EGFP and TagRFP-T fluorophores since it is a single molecule.

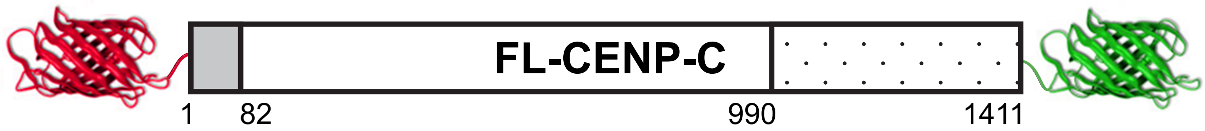

2) Quantify relative intensities of (background corrected) EGFP and TagRFP-T signals to get a correction ratio.

(Example: EGFP to TagRFP-T correction ratio = 3.5)

3) Image TagRFP-T-CENP-C-TR + VH-EGFP with the same imaging parameters as above (step 1) and apply the correction ratio (step 2)

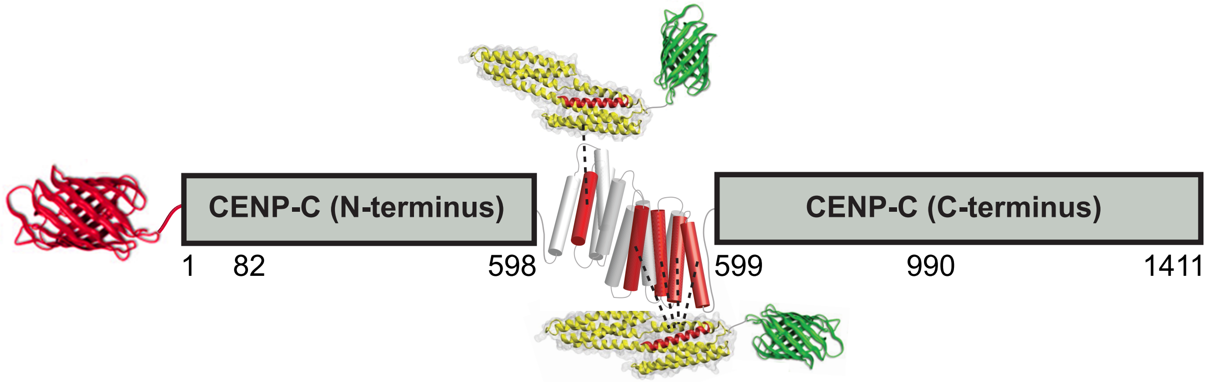

(Example: If the background corrected ratio of VH-EGFP to TagRFP-T-CENP-C-TR at the kinetochores is 7 then this value divided by the correction ratio of 3.5 yields 2 VH molecules per TR)

Supplementary Figure 2. Basic outline of the method used to count the number of VH molecules bound per CENP-C-TR. Further details are provided in the materials and methods.

Supplementary Figure 3

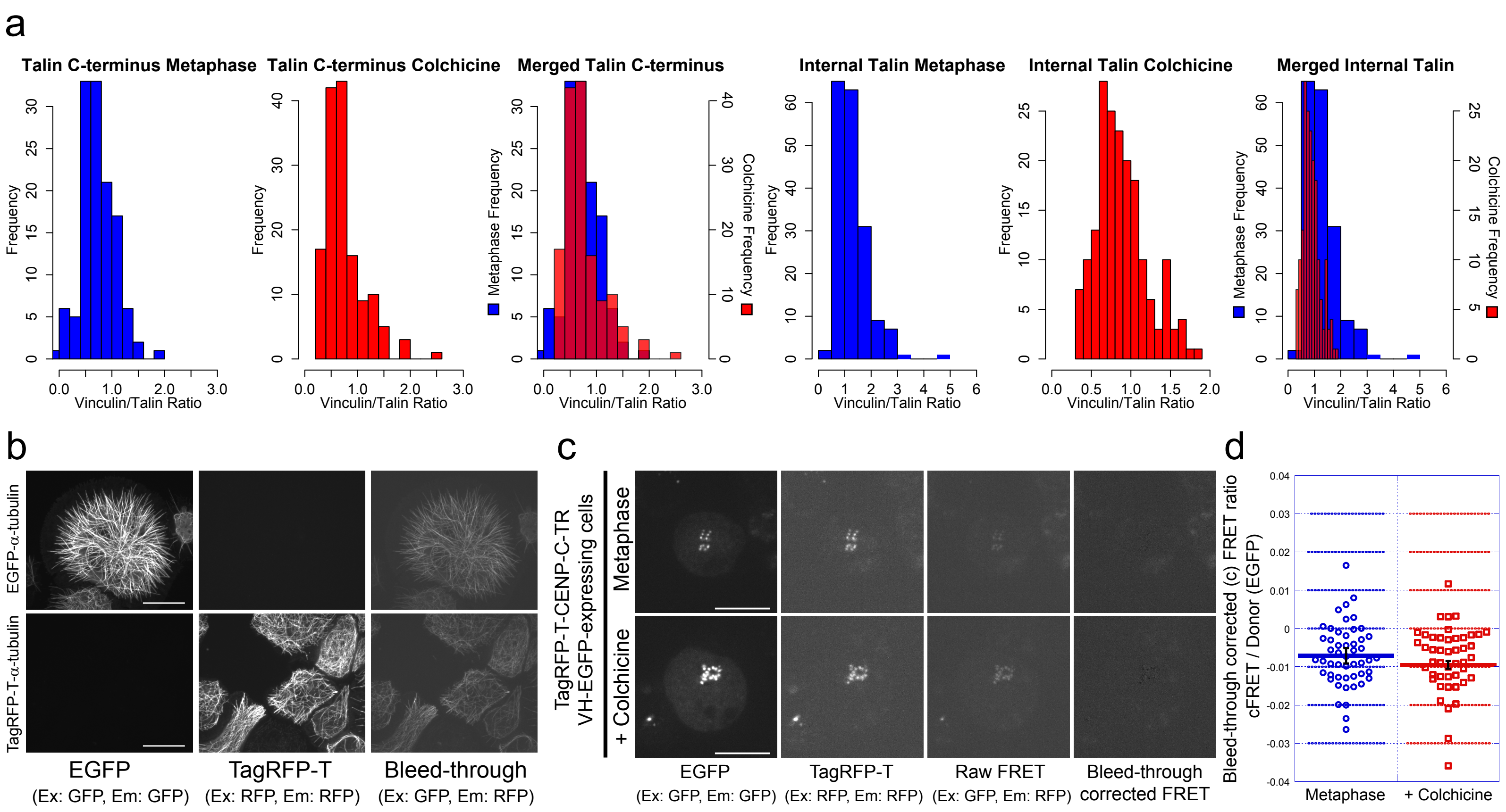

Supplementary Figure 3. (a) Histograms showing the distributions of VH per TR measurements for the internal and C-terminal CENP-C-TR reporters at unattached and bioriented metaphase kinetochores. The blue and red histograms represent metaphase and unattached measurements respectively. The third and sixth columns contain merged metaphase and unattached histograms for the C-terminal and internal reporters. Other than the C-terminal TR reporter in metaphase, the data exhibited a non-normal distribution as determined by the Shapiro-Wilk Normality test in R. Thus, all reported p-values for these data are from the Mann-Whitney-Wilcoxon test. (**b**) TagRFP-T-α-tubulin and EGFP-α-tubulin expressing cells were imaged to quantify spectral bleed-through from EGFP and TagRFP into the FRET channel on the spinning disk confocal microscope used to investigate if there was FRET between TagRFP-CENP-C-TR and VH-EGFP. Comparable channels are displayed with identical contrast and brightness scaling. The imaging conditions yielded ~6% and 12% spectral bleed-through from EGFP and TagRFP-T respectively. (**c**) Representative images of metaphase and colchicine-treated cells co-expressing TagRFP-T-CENP-C Internal TR and VH-EGFP imaged under the identical conditions as were used to measure the spectral bleed-through into the FRET channel. Pixel-by-pixel subtraction of the bleed-through in Metamorph yields no detectable FRET (Bleed-through corrected FRET images) between the CENP-C-TR and the VH. (**d**) Bleed-through corrected (c) FRET ratios (cFRET / background corrected EGFP signal) for metaphase and colchicine treated cells co-expressing TagRFP-T-CENP-C Internal TR and VH-EGFP. No FRET signal is detectable. n = 50 centromeres/kinetochores from 10 metaphase cells, n = 48 centromeres from 10 colchicine-treated cells. Scale bars are 10 μm. Error bars are SEM

Supplementary Figure 4

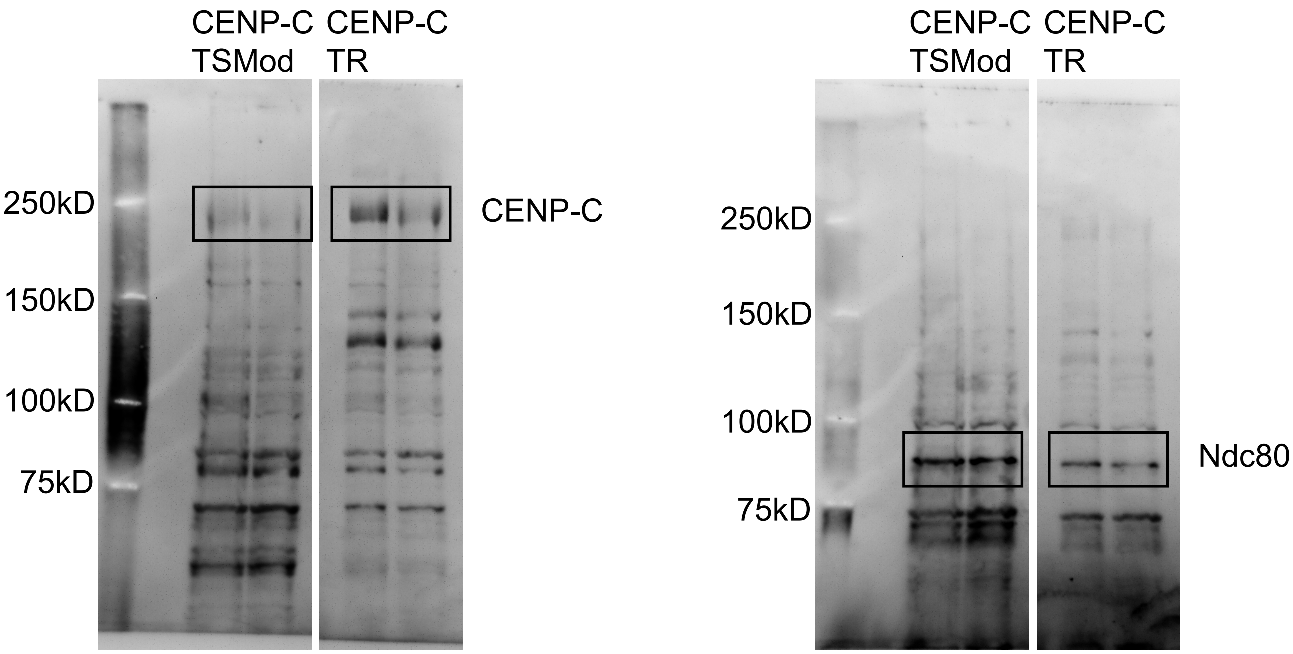

Supplementary Figure 4. Uncropped western blots with the cropped images shown in the main text highlighted in the boxes.
